# How to make a static cytokinetic furrow out of traveling excitable waves 

**DOI:** 10.1080/21541248.2016.1168505

**Published:** 2016-04-12

**Authors:** Andrew B. Goryachev, Marcin Leda, Ann L. Miller, George von Dassow, William M. Bement

**Affiliations:** a Centre for Systems and Synthetic Biology, Cell Biology Institute, School of Biological Sciences, University of Edinburgh, Edinburgh, UK; b Department of Molecular, Cellular and Developmental Biology, University of Michigan, Ann-Arbor, MI, USA; c Oregon Institute of Marine Biology, University of Oregon, Charleston, OR, USA; d Laboratory of Cell and Molecular Biology, Graduate Program in Cell and Molecular Biology, University of Wisconsin-Madison, Madison, WI, USA

**Keywords:** activator-inhibitor coupling, cytokinesis, Ect2, excitable dynamics, F-actin, guanine nucleotide exchange factor, Rho small GTPases, Rho zone, traveling waves

## Abstract

Emergence of the cytokinetic Rho zone that orchestrates formation and ingression of the cleavage furrow had been explained previously via microtubule-dependent cortical concentration of Ect2, a guanine nucleotide exchange factor for Rho. The results of a recent publication now demonstrate that, en route from resting cortex to fully established furrow, there lies a regime of cortical excitability in which Rho activity and F-actin play the roles of the prototypical activator and inhibitor, respectively. This cortical excitability is manifest as dramatic traveling waves on the cortex of oocytes and embryos of frogs and starfish. These waves are initiated by autocatalytic activation of Rho at the wave front and extinguished by F-actin-dependent inhibition at their back. It is still unclear how propagating excitable Rho-actin waves give rise to the stable co-existence of Rho activity and F-actin density in the static cleavage furrow during cytokinesis. It is possible that some central spindle-associated signaling molecule simply turns off the inhibition of Rho activity by F-actin. However, mathematical modeling suggests a distinct scenario in which local “re-wiring” of the Rho-actin coupling in the furrow is no longer necessary. Instead, the model predicts that the continuously rising level of Ect2 produces in the furrow a qualitatively new stable steady state that replaces excitability and brings about the stable co-existence of high Rho activity and dense F-actin despite the continuing inhibition of Rho by F-actin.

Small GTPases of the Rho family are well established as the drivers of cellular morphogenesis.^1^ Activation of Rho GTPases frequently serves as the pre-pattern that guides formation of various cellular structures, such as yeast bud,^2^ animal cell apical domain,^3^ filopodia^4^ and podosomes.^5^ Of these structures, the cytokinetic actomyosin contractile ring^6^ is, perhaps, the most important for the very viability of animal cells, and its positioning, molecular architecture and mechanism of contractility have been a matter of active research for several decades.^7-9^ In animal cells, RhoA is a key player in the establishment of the cytokinetic ring, and its local activation in the shape of an equatorial cortical belt, dubbed the Rho zone,^10,11^ was shown to be the direct precursor of the nascent actomyosin ring and cytokinetic furrow. Not surprisingly, fluorescence microscopy readily shows strong signals for both F-actin and active Rho at the cytokinetic furrow throughout ingression.^12^ Rho directs formation and constriction of the actomyosin ring primarily via its effectors, formins and Rho kinases (ROCKs), which polymerize F-actin and activate myosin-2 motors, respectively.^7^ Other Rho effectors, such as anillin^13^ and citron kinase,^14^ also contribute to contractile ring constriction. Rho and F-actin are closely associated in the context of other cellular processes, for example, formation of stress fibers,^15^ closure of cellular wounds,^16^ regulation of apical cell-cell junctions,^17,18^ and bleb retraction.^19,20^ This apparent association between Rho and F-actin is so widespread that it is not uncommon to think of them as co-operating partners. But are the dynamics of Rho activation and F-actin polymerization invariably parallel?

A recent paper^21^ from our groups shows that the dynamic relationship between Rho and F-actin can be unexpectedly complex. Remarkably, while Rho was found to promote F-actin polymerization (as expected), F-actin, surprisingly, was found to inhibit Rho. Furthermore, we concluded that this relationship formed the molecular basis of a paradigmatic activator-inhibitor mechanism. The significance of this conclusion stems from the fact that theoretical efforts have repeatedly shown that activator-inhibitor mechanisms underlie formation of traveling waves of excitable activity^22,23^ as well as static Turing-type structures,^24-26^ but strong empirical support for these notions has been scant. In 1952, in an effort to explain the physical principles underlying biological morphogenesis, Alan Turing postulated an elegant mechanism explaining the *de novo* emergence of a stable pattern from a spatially homogeneous state.^24^ In this reaction-diffusion mechanism, an autocatalytically reproducing activator generates its own inhibitor that limits the spread of the activator (see Fig. 1A). For the existence of Turing-type structures, which usually take the form of spots, stripes and labyrinthine patterns, it is crucial that the inhibitor diffuses much faster than the activator. This fast removal of the inhibitor by the diffusive transport permits the activator to avoid the complete suppression by the inhibitor and allows the activator to accumulate to a sustainable level within the structure's core (see Fig. 1C).

**Figure 1.**
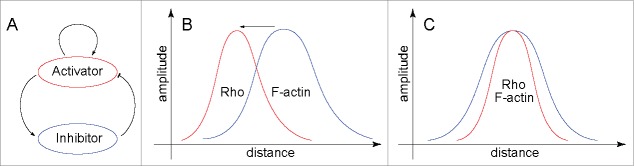
Spatial profiles of the Rho activity and F-actin accumulation are distinct in a traveling wave and a static structure. (A) Relationships between the prototypical activator and inhibitor shown here schematically can generate both waves of excitable dynamics and static structures. (B) In a schematic wave spatial profile, the activator (Rho) always precedes the inhibitor (F-actin). (C) In an immobile structure, such as the cytokinetic furrow or Turing pattern, the activator and inhibitor peak together. The maxima of Rho and F-actin are scaled to be equal. The direction of wave propagation in (B) is shown by the arrow.

## Cortical waves of Rho activity are followed by inhibitory waves of F-actin

As is often the case, this work began with a serendipitous discovery: experimental overexpression of Ect2, a conserved guanine nucleotide exchange factor (GEF) for Rho, elicited high-amplitude cortical Rho waves in oocytes and embryos of frogs and starfish. This Rho GEF, also known as Pebble in *Drosophila*, is responsible for the activation of Rho in the context of cytokinesis in many model organisms.^7^ In the initial experiments, Ect2 overexpression was employed to enhance the cytokinetic Rho zone. Surprisingly, the fluorescent reporter for Rho activity^16^ detected not only an amplified Rho zone but also revealed fainter yet unmistakable waves of active Rho throughout the cortex. Follow-up experiments demonstrated that cortical waves of Rho activity are a normal feature of frog and starfish oocytes and embryos, as are waves of F-actin polymerization and de-polymerization. As treatment with a bacterial toxin C3 transferase that inhibits Rho by ADP-ribosylation or the expression of a dominant-negative Ect2 mutant both erased the F-actin waves, it became clear that Rho activity was upstream of the initiation of the F-actin waves.

The fact that F-actin waves followed behind and partially overlapped the waves of Rho activity suggested that actin polymerization somehow contributed to extinguishing the Rho activity. This hypothesis was directly confirmed experimentally: localized depolymerization of actin with latrunculin, which prevents F-actin polymerization by sequestering monomeric G-actin, caused a co-localized increase in the amplitude and wavelength of Rho activity waves. Kinetic analysis of live-cell imaging data further confirmed that F-actin inhibits Rho activity. Significantly, this analysis also showed that Rho exhibits autocatalytic self-activation at the front of the waves and induces the accumulation of F-actin. Thus, experimental manipulation and theoretical data analysis together established that Rho activity and F-actin accumulation formally satisfied the requirements for the prototypical activator and inhibitor, respectively. Prior to the Bement et al. paper,^21^ F-actin had been conjectured an inhibitor of lamellipodia protrusion in fibroblasts by Vavylonis and colleagues,^27^ but no direct relationship with the activity of a particular small GTPase had been demonstrated. Taken together, the results of both studies suggest that F-actin accumulation could potentially inhibit the activity of some members of the Rho family in the context of several cellular processes. However, the molecular details of this likely indirect inhibition remain a subject for future studies.

## Why is Rho activity not suppressed by F-actin in the furrow?

If F-actin indeed inhibits the activity of Rho, how can an immobile cytokinetic Rho zone co-exist with propagating Rho-actin waves in the same cell? That this co-existence is an apparent paradox can be seen from the following arguments. A main tenet of excitable dynamics posits that the very existence of an excitable wave is dependent on its ability to propagate with a constant velocity into the space free of the inhibitor. The wave propagates because the activator perpetually “runs away” from the inhibitor that the activator generates itself (see Fig. 1B).

Why then does the furrow not move along the cortex? The fact that it does not normally do so implies that the furrow cannot be a wave. Following furrow initiation, distinct waves are no longer evident and a relatively stable Rho zone is formed. A comparison of the spatial profiles of Rho and F-actin in the static furrow versus the waves shows that, while in the propagating wave (Fig. 1B) the spatial profile of the activator (Rho) always peaks ahead of the inhibitor (F-actin), in the static cytokinetic furrow (Fig. 1C) the peaks of Rho and F-actin always spatially coincide.^12^ When the diffusivity of the inhibitor is substantially higher than that of the activator, the activator-inhibitor system can form static Turing-type structures with coinciding peaks of the activator and inhibitor. Therefore, the furrow could, in principle, be such a Turing structure. This hypothesis faces at least 2 challenges. First, the cortical diffusivity of the active Rho is unlikely to be smaller than that of the polymeric F-actin, whose diffusivity is arguably negligible. Second, Turing structures, once formed, are expected to be self-sustaining. Depolymerization of spindle microtubules prevents formation of the furrow and also was seen to reverse the ingression of the already formed furrows.^28^ In our experiments, microtubule depolymerization rapidly extinguished the activity of Rho in the established equatorial Rho zone of a starfish embryo.^21^ Therefore, the Rho zone and the entire cytokinetic furrow are not fully sustainable in the absence of microtubules. Thus, the furrow with its coinciding peaks of Rho activity and F-actin density is neither a traveling wave nor a static Turing-like structure and cannot be explained by the activator-inhibitor model alone without additional mechanisms, extrinsic to the activator-inhibitor system, such as the contribution of the spindle microtubules. Furthermore, the co-existence of a furrow and excitable Rho-actin waves within the same cell suggests that, in the furrow, the relationship between Rho and F-actin is somehow distinct from that in the rest of the cell cortex.

Which factors could make the cortical properties in the furrow distinct from those in the rest of the cell cortex? These factors are likely to be the same as the ones responsible for the formation of the furrow in the first place. According to the current paradigm, the mitotic spindle is responsible for the induction of the cytokinetic Rho zone by concentrating Ect2 on the equatorial cortex via interaction of Ect2 with the centralspindlin complex. Centralspindlin, comprised of a plus-end-directed kinesin (MKLP1) and a Rho GAP protein (MgcRacGAP), accumulates on the overlapping microtubules of the central spindle.^29^ Thus, one potential explanation for the co-existence of excitable waves with a static furrow could be that some signaling molecule(s), whose activity is confined to the narrow equatorial region of the cell by the central spindle microtubules, could alter the wiring of the cortical network of biochemical reactions, e.g., by turning off the inhibition of Rho activity by F-actin. For example, it is well-established that 2 important mitotic kinases, Aurora B^30^ and Plk1,^31^ localize to and are active at the central spindle during cytokinesis. It is thus conceivable that phosphorylation by one or both of these kinases could alter the activator-inhibitor coupling between the Rho activity and F-actin accumulation to enable their stable co-existence in the furrow.

## Mathematical model offers a potential explanation

**Figure 2.**
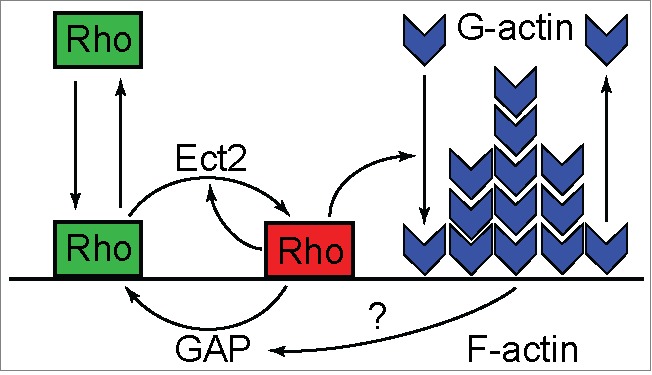
Scheme of the biochemical reactions described by the mathematical model of excitable Rho dynamics (adapted from Ref. 21). Inactive Rho (green rectangles) shuttles between the membrane and the cytoplasm and becomes converted into the active form (red rectangle) by Ect2. Active Rho promotes actin polymerization that, in turn, facilitates Rho inactivation, possibly, by recruiting a Rho GAP. In the absence of Rho activity, F-actin depolymerizes and recycles back into the cytoplasm.

**Figure 3.**
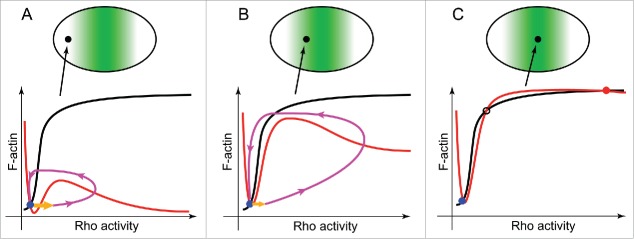
Mathematical model predicts that the type of cortical dynamics observed in dividing cells is determined by the local concentration of the Rho GEF Ect2. Cortical loci positioned along the division axis of a cell (upper row) are differentiated by the local concentration of Ect2 that increases toward the cell equator and exhibit distinct types of the local Rho-actin dynamics schematically shown by the characteristic phase portraits (bottom row). (A) Weak excitability far from the nascent furrow. (B) Well-developed excitable waves in the vicinity of the furrow. (C) High Rho activity and F-actin accumulation stably co-exist in the furrow where a new stable steady-state emerges due to the high local concentration of Ect2. Local concentration of Ect2 at the cortical loci indicated by filled black circles is shown schematically by the intensity of green shading (upper row). Shown on the phase portraits (bottom row): red curves – the activator nullclines; black curves – the inhibitor nullclines; magenta curves – excitable trajectories; orange arrows – initial perturbations required to induce excitable response; blue filled circles – the resting cortical steady state; red filled circle – the high activity cortical steady-state in the furrow; black open circle – the unstable steady state. See text for discussion.

Surprisingly, analysis of the mathematical model describing excitable dynamics of Rho-actin waves suggested an alternative hypothesis that explained the simultaneous presence of propagating waves and static cytokinetic Rho zone without the need to change the wiring of the reaction network. The model captures only the essential interactions within the Rho-actin network (see Fig. 2) in a simplified heuristic approach that is based on the previous model of a Rho-family GTPase nucleotide cycling coupled to its membrane-cytoplasmic shutling.^32^ It describes the spatio-temporal behavior of the membrane pools of the active GTP-bound Rho (RT), inactive GDP-bound Rho (RD), and F-actin (F) by the system of 3 coupled reaction-diffusion equations. RD is converted to RT by the reaction of activation, which is assumed to be nonlinear, autocatalytic and mediated by Ect2, while RT is converted to RD by inactivation, which is both constitutive and actin-induced. RD is allowed to freely exchange with a pool in the cytoplasm, which, as a simplification, is assumed to be constant. Ignoring diffusion and considering only the reaction terms, the temporal evolution in one spatial location of the cell cortex can be represented by a trajectory in the space of the 3 chosen variables (RT, RD, F). To further simplify the description, this trajectory can be projected onto the activator-inhibitor phase plane (RT, F) as shown in Figure 3. On such a phase portrait, the positions where either RT or F do not change with time are given by the so-called nullclines, the curves where (red curves on Fig. 3) or (black curves on Fig. 3), respectively. Since in a steady-state, by definition, all system variables do not change, all possible steady states of the model must lie on the intersection of the 2 nullclines. Excitable behavior of the activator-inhibitor model can be illustrated by a typical phase trajectory shown in Figure 3A by the magenta curve. In response to a super-threshold perturbation (orange arrow), an excitable system leaves its position of rest at the steady-state and performs a burst of activity throughout which first the activator and then the inhibitor peak prior to eventually returning back to the steady state. The amplitude and the shape of this activity burst are primarily determined by the nonlinear behavior of the activator, RT.^23^ On the phase plane (RT, F), this behavior is graphically represented by the sigmoid-shaped activator nullcline. How could this model help explain the experimental observations?

In a dividing starfish blastomere, Rho-actin waves suddenly emerge throughout the cell cortex at the onset of anaphase but then rapidly disappear from the poles and gather toward the cell equator with the concomitant increase in their amplitude. As the area occupied by waves continues to narrow, it acquires the typical appearance of the static cytokinetic Rho zone in which waves can no longer be distinguished. These results suggest that the transition from the spatially distributed excitable dynamics to the static furrow reflects accumulation of Ect2 on the equatorial cortex due to the transport activity of the centralspindlin complex. The model captures this behavior and mathematically explains the transition from waves to furrow. Indeed, when observed in the middle of this transition, starfish blastomeres exhibit a strong gradient of Rho-actin dynamics from the poles to the equator. These spatially-dependent cortical dynamics can be represented by a sequence of phase portraits corresponding to the model behavior at the progressively increasing levels of Ect2, as shown in Figure 3. Near the cell poles, the local cortical concentration of Ect2 is low (Fig. 3A), and the model has only one steady-state (blue dot) that corresponds to the “non-excited” state of the cortex with a negligible level of Rho activity. A large perturbation is required to produce even a modest excitable response so that, in the actual experiment, no excitable dynamics may be seen at all in this cortical area of weak excitability. Closer to the cell equator (Fig. 3B), the threshold of excitation is lower and the amplitude of excitable dynamics is larger. Here robust excitable waves can be readily seen traversing the cell cortex. Further increase in the local Ect2 concentration, beyond a certain critical value, causes the rising activator nullcline to intersect the inhibitor nullcline as shown in Figure 3C. Two new steady states are born at this point, a new stable steady-state (red dot) with high Rho activity and F-actin accumulation, and an unstable state (black circle). At this configuration of the nullclines, the cortex is formally bistable but, in practice, any significant perturbation (e.g., the one provided by a propagating excitable wave,) will push the system into the high activity steady state. If Ect2 concentration is increased even further, this will result in a nullcline configuration in which only the high activity steady-state is possible (not shown). However, regardless of whether the cortex is bistable or monostable with the high activity state, it ceases to be excitable and stops supporting traveling waves. Instead, its state corresponds to the experimentally observed static furrow whose width is then predicted to be determined by the width of the equatorial cortex where the Ect2 concentration is above the critical. The model further predicts that since the total cellular Ect2 content is conserved, a spatial redistribution of Ect2 resulting from the depolymerization of microtubules should restore uniform cortical wave dynamics. This is indeed the case as was demonstrated by us experimentally using a starfish zygote that had formed a furrow prior to its treatment with nocodazole that rapidly disassembled spindle microtubules; after the treatment, uniform cortical waves were restored. Additionally, the model predicts that if a cell overexpresses Ect2, its focusing into the furrow during cytokinesis need not deplete the rest of the cortex to the point of complete disappearance of waves. This behavior is indeed seen in Ect2 overexpressing frog blastomeres, which form prominent furrows without stopping to support wave propagation outside the furrows.

In conclusion, the model predicts that simply by gradually increasing Ect2 concentration at the equatorial cortex at the expense of the rest of the cell, it is possible to convert a resting “non-excited” cortex into a full-fledged cytokinetic furrow via the excitability route. This prediction is non-trivial since, according to the model, even within the static furrow the Rho activity and F-actin accumulation remain coupled as the activator and inhibitor. Due to the emergence in the furrow of a new stable steady state, the Rho activity is not extinguished by the F-actin, and the furrow need not propagate like a wave to sustain this activity. Further work will show whether this prediction is correct or local “re-wiring” of the interactions between Rho and F-actin is required to enable their stable co-existence within the furrow.
